# A Chinese scoring system for predicting successful retrograde collateral traverse in patients with chronic total coronary occlusion

**DOI:** 10.1186/s12872-023-03405-6

**Published:** 2023-07-29

**Authors:** Qiu Yu Li, Xiao Long Lin, Fan Qi Li, Zi Chao Cheng, Jia Yu Tian, Dong Hui Zhao, Wayne Bond Lau, Jing Hua Liu, Qian Fan

**Affiliations:** 1 Center for Coronary Artery Disease, Beijing Anzhen Hospital, Capital Medical University, and Beijing Institute of Heart, Lung, and Blood Vessel Diseases, Beijing, 100029 China; 2 Department of Emergency Medicine, Thomas Jefferson University, Philadelphia, PA 19107 USA

**Keywords:** PCI, Chronic total occlusion, Retrograde approach, Scoring system

## Abstract

**Background:**

Retrograde approach technique has been challenging in percutaneous coronary interventional treatment of chronic total occlusion (CTO) coronary disease. The present study endeavors to determine a novel Chinese scoring system for predicting successful collateral channels traverse via retrograde approach.

**Methods:**

The demographic characteristics and angiographic characteristics of 309 CTO patient were analyzed by univariable and multivariable analysis for selecting potential predictors. And the nomogram was used to establish the scoring system. Then it was evaluated by the internal and external validation.

**Results:**

The predictors of Age, Connections between collateral channels and recipient vessels, and Channel Tortuosity (ACT) were identified with univariable and multivariable analysis and employed to the ACT score system. With acceptable calibrations, the area under curve of the scoring system and the external validation were 0.826 and 0.816 respectively. Based on score, the predictors were divided into three risk categories and it showed a consistent prediction power in the validation cohort.

**Conclusions:**

The novel Chinese ACT score is a reliable tool for predicting successful retrograde collateral traverse.

**Supplementary Information:**

The online version contains supplementary material available at 10.1186/s12872-023-03405-6.

## Background

As modernization of diet has occurred in the past decades, the prevalence of coronary disease in the Chinese population has increased annually. More than 1000,000 percutaneous coronary intervention (PCI, a main therapy of coronary disease) procedures are conducted per year in China, a value projected to increase by 100,000 annually [1]. Within the complex coronary disease umbrella, chronic total occlusion (CTO) remains the most challenging subtype to treat successfully. The success rate for PCI in the treatment of other complex coronary diseases (such as left main coronary artery diseases, coronary calcifications, and coronary bifurcation lesions) is nearly 100%. In contrast, PCI success rate for CTO is only 60% [2]. PCI done for CTO is additionally high risk, and of long duration. As a result, CTO patients are more likely to choose coronary bypass surgery for treatment. CTO itself significantly decreases myocardial blood supply and is a major cause of sudden cardiac death and ischemic heart failure [3].

An important therapy has been developed to treat CTO, given its unique pathophysiologic characteristics [4, 5]. Retrograde intervention employs a technique in which a guide wire is fed antegrade via collateral channel, and passes through the occluding lesion in retrograde, backwards fashion to reach the proximal occlusion [6]. This approach has greatly augmented procedural success rates, has received international general recognition as the main procedure for CTO treatment, and is regarded as an effective complement to existent forward-moving interventional techniques [7].

Many independent predictors and scoring systems (derived from retrospective clinical case analyses) exist to grade interventional surgical techniques. However, most of those score systems (such as the J-score, PROGRESS-CTO, and CL-score) were designed for antegrade interventional measures, and therefore only apply to forward-moving approaches [8–14]. Few scoring systems apply strictly to retrograde interventional techniques [15]. To our knowledge, there is no validated score system predicting the successful traverse of collateral channels by retrograde interventional technique. The sheer number of procedures performed in China affords unique opportunity to investigate the factors influencing the success of collateral circulation crossing in retrograde CTO-PCI cases.

## Methods

### Ethics approval and consent to participate

The research ethics committee of the Beijing Anzhen Hospital in China approved this study and waived informed consent. All the participants agreed with this study and signed the informed consent. The study was carried out in accordance with Helsinki Declaration.

### Study population

The study population included 9841 patients received PCI treatment in the Center for Coronary Artery Disease, Beijing AnZhen Hospital during June 2015 to December 2021. Total 1508 consecutive patients with CTO lesions we diagnosed. Among them, 309 CTO patient underwent PCIs via retrograde approach were finally included.

### Collateral channels grouping

**Fig. 1:**
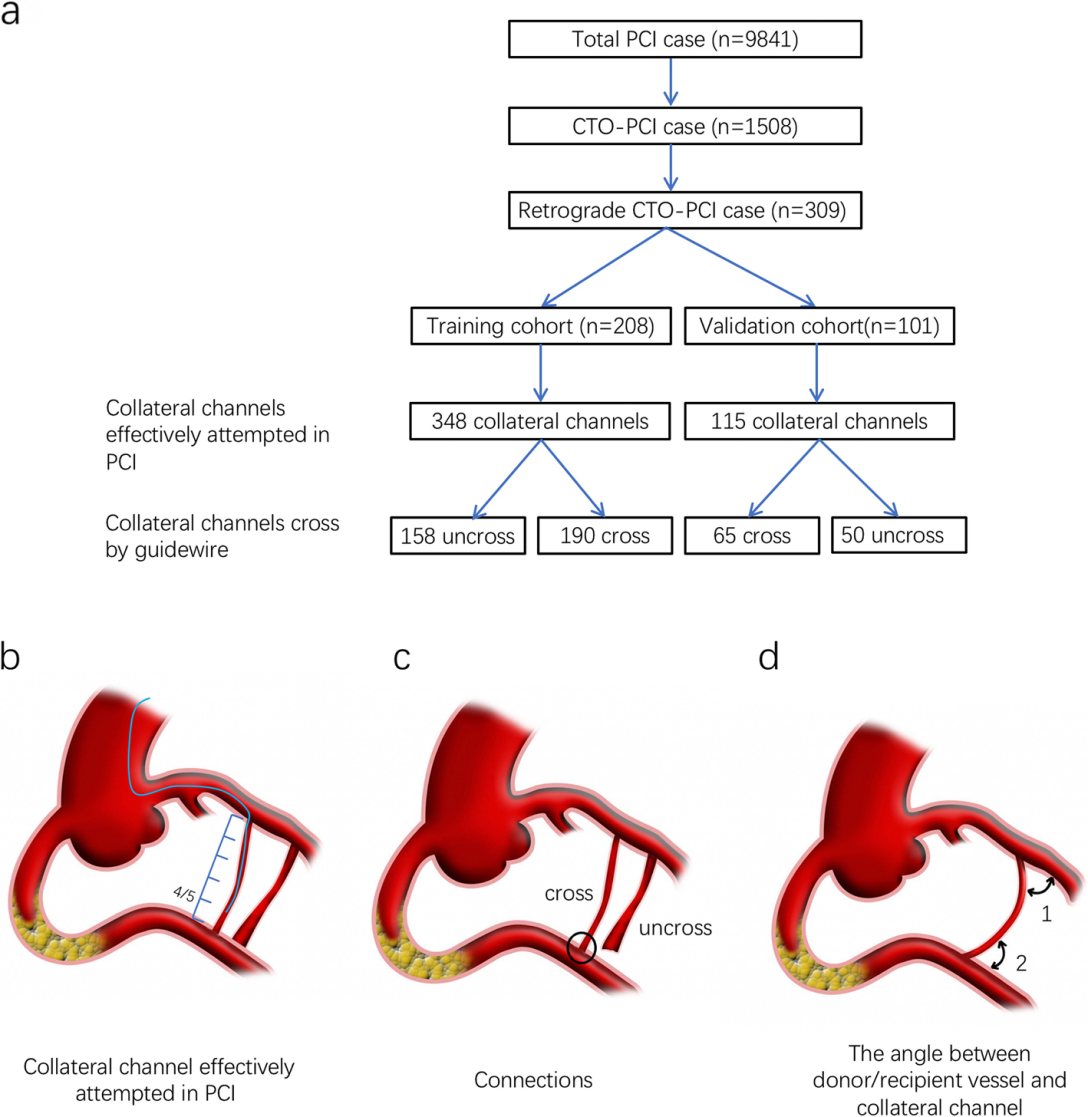
Study protocol and definition: **a** Total 9841 patients received PCI treatment in our institution. 1508 total patients with chronic total occlusion (CTO) were treated by percutaneous coronary intervention (PCI). Of these patients, 309 total patients underwent retrograde PCI for CTO recanalization (1199 patients were treated via antegrade approach only). They were divided into a training cohort (208 patients with 348 collateral channels) and a validation cohort (101 patients with 115 collateral channels); **b** An effective attempt was defined as the guide wire tip traveling over 4/5th the distance from donor to recipient vessels; **c** Connections between collateral channels and recipient vessels; and **d** Angle between collateral channels and donor (or recipient) vessels

All the clinical and angiographic data from 309 patients with total 458 collateral channels attempted in the PCI were used to establish a database. Only the collateral channels that were effectively attempted were collected in the analysis. Collateral channels were considered effectively attempted when the guide wire tip traveled over 4/5th of the distance from the donor to the recipient vessels. Between June 2015 and December 2019, an independent cohort of 208 consecutive patients with 348 collateral channels formed the training cohort. Between January 2020 to December 2021, an independent cohort of 101 consecutive patients with 115 collateral channels formed the validation cohort for this study. Two cohorts were with no patient overlap. According to whether guidewire passage through collateral channels and successfully reached recipient vessels, the 458 collateral channels in each cohort were divided into collateral channels traverse success group and collateral channels traverse failure group (Fig. 1a).

### Interventional procedures

Interventional cardiologists with more than 10 years of experience in CTO-PCI performed all procedures. Clinical decisions for each case were made completely at the physician operator’s discretion. All patients were pretreated with aspirin and clopidogrel or ticlopidine. Weight-adjusted heparin or bivalirudin was administered to maintain activated clotting time exceeding 300 s, which was monitored per 30-min intervals. The guide catheters (7 Fr size) were introduced for both CTO and at the donor site of the coronary arteries. To evaluate CTO lesion morphology, bilateral simultaneous coronary injections were performed. A dedicated guide wire (SION) over a microcatheter (Corsair or Finecross) was inserted through the favorable collateral channel to reach the distal CTO segment, where retrograde CTO crossing was performed with either softer polymer jacket wires or stiffer CTO guide wires. Both sequence of wiring technique and guide wire selection were dependent upon patient coronary anatomy and operator discretion. The reverse-CART technique was employed in some patients with simultaneous antegrade and retrograde subintimal tracking to limit dissection to the CTO site for successful recanalization.

### Definitions

CTO is defined as a lesion demonstrating a TIMI flow grade of 0 for at least 3 months. The duration of occlusion is estimated by symptom onset, history of angina, or previous myocardial infarction in the same territory or demonstrated by previous angiography.

A collateral channel effectively attempted in PCI is defined as the guide wire tip traveling over 4/5^th^ the distance from donor to recipient vessels in the PCI procedure (Fig. 1b).

Successful retrograde collateral traverse (collateral channel cross) is defined as guidewire passaging through collateral channel that successfully reached recipient vessels.

Procedural success is defined as residual stenosis of less than 30% with TIMI flow grade 3 and without complications which encompass collateral perforation, side branch occlusion, donor vessel dissection, and recipient vessel dissection.

Severe calcification is identified as radiopacities seen without cardiac motion before contrast injection, usually affecting both sides of the arterial lumen [16]. Presence of calcification was assigned to 1 of 2 categories according to severity (none or severe).

Bending is defined as at least 1 bend of ≥ 45° (or < 45°) assessed by angiography throughout the occluded segment divided into either CTO entry or CTO body.

The size of collateral channel diameter is assessed by 3 grades: CC grade 0 (no continuous connection between donor and recipient artery); CC grade 1 (continuous, threadlike connection, diameter ≤ 0.4 mm); and CC grade 2 (continuous, small branch-like vessel throughout its course, diameter > 0.4 mm) [16]. Rentrop grades were assigned per the following: Rentrop Grade 0 (no clear filling of any collateral channels); Rentrop Grade 1 (collateral filling of vessel branches dilated without any dye reaching the epicardial segment of that vessel); Rentrop Grade 2 (partial collateral filling of the epicardial segment of the vessel being dilated); Rentrop Grade 3 (complete collateral filling of the vessel being dilated).

Connections between collateral channels and recipient vessels (abbreviate for Connections, Fig. 1C, circled), are divided into 2 grades: clear or ambiguous/invisible.

**Fig. 2:**
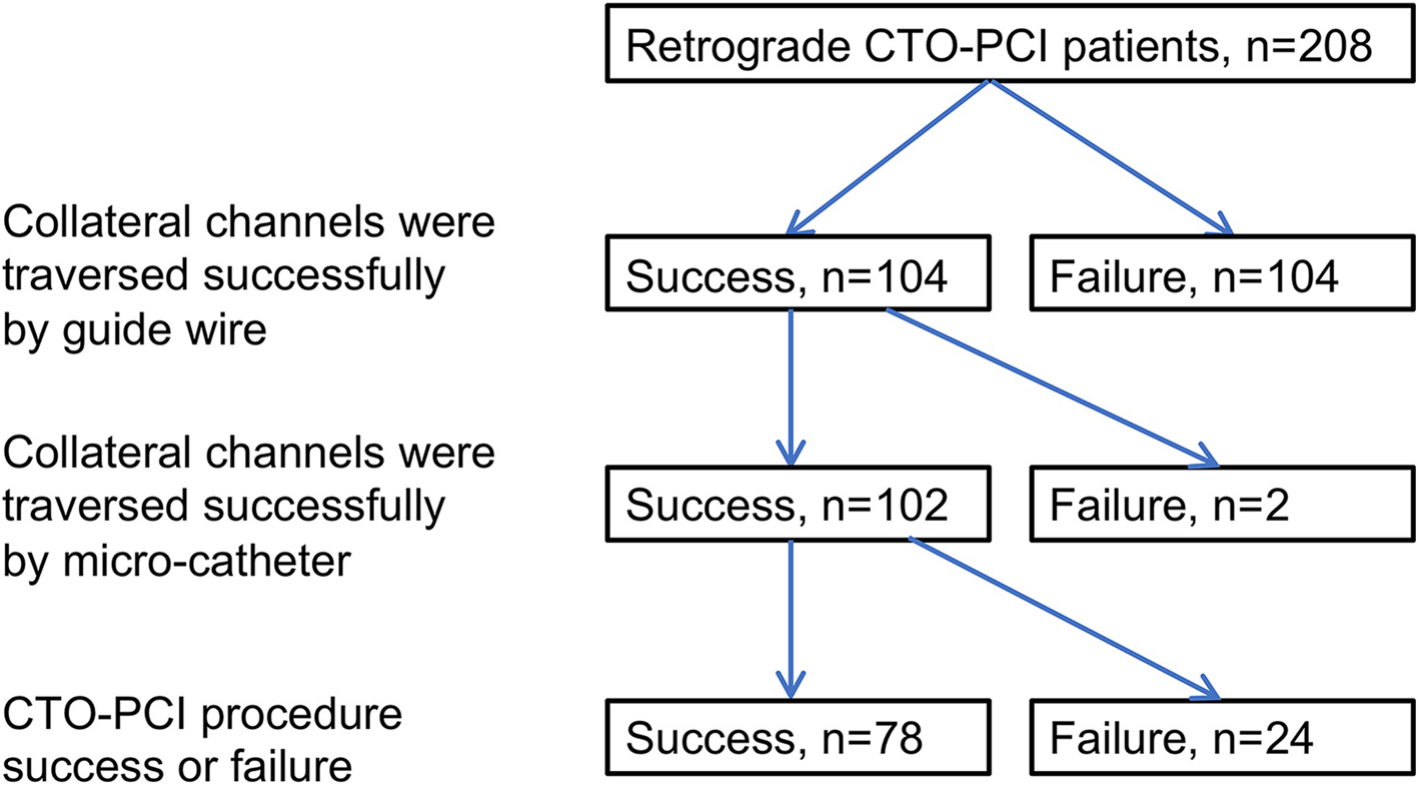
Procedural techniques and population characteristic in the training cohort: The collateral circulation was successfully traversed by both guide wires and catheters in 102 cases. Successful intervention was achieved via retrograde approach without any complications in 78 cases

The angle between donor vessels and collateral channels (Fig. 1d-1), and the angle between recipient vessels and collateral channels (Fig. 1d-2) have been delineated in Fig. 1.

Collateral channels tortuosity is defined angiographically, as a target lesion bend exceeding 45˚. Furthermore, collateral tortuosity is grouped into the following types. Tortuosity less than 90° is termed mild, defined as making up to 1 complete half circle, and straightening subsequently thereafter. Tortuosity exceeding > 90° is termed severe/corkscrew and defined as making 1 circular bend simultaneously during the whole course.

### Data collection

The demographic characteristics, CTO lesion angiographic characteristics, collateral channel angiographic characteristics, and CTO-PCI complications were collected by electronic case report forms and analyzed retrospectively by at least 2 senior cardiologists. 309 patients with 458 total collateral channels efficiently attempted in PCI were collected. The demographic characteristics included sex, age, body mass index (BMI), history of smoker, hypertension, hyperlipemia, diabetes mellitus, medicine before PCI, Left ventricular ejection fraction (LVEF). The CTO lesion angiographic characteristics included targeted vessel, in-stent occlusion, morphology of entry point, calcification of lesion, occluded route bending, proximal cap side-branch, and occluded period. The collateral channel angiographic characteristics collateral channels types, CC grade, Rentrop score, Connections, angle between donor vessels and collateral channels, angle between receptor vessels and collateral channels and channel tortuosity. The CTO-PCI complications included collateral perforation, side branch occlusion, donor vessel dissection, and recipient vessel dissection.

### Statistical analysis

In the current study, we used 309 CTO patients as demographic characteristic analysis subject and 463 collateral channels effectively attempted in the PCI as angiographic characteristic analysis objects. Categorical data were described as the number (percentage). Statistical analysis in the baseline was performed via Chi-square test for comparison of categorial variables. Fisher's Exact test was used for the comparison of complication rate of retrograde CTO-PCI in septal and epicardial collateral channels. The values in the clinical practice that were difficult to be categorized in some variables (including Occluded period, CC score, Rentrop score, Connection, Angle between donor vessels and collateral channels, Angle between receptor vessels and collateral channels, and Channel tortuosity) are treated as missing values. In the univariable regression analysis, cases with missing values for that variable were excluded. In the multivariate regression analysis, cases with missing values for any variable were also excluded. To determine independent predictors of retrograde PCI success, all the differences in demographic characteristic and CTO lesion characteristic were analyzed by univariable logistic regression analysis. The differences of multiple collateral characteristics measurements per person were analysis by estimated by Generalized Estimating Equations. Multivariable logistic regression analysis was performed on the variables with significant differences in the univariable regression and Generalized Estimating Equations analysis. Potential predictors of successful retrograde collateral channels traverse were retained for nomogram predictive scoring system building. The training cohort data were used for the discrimination and calibration of the scoring system. Finally, the verification cohort was used for exam the predictive value of the scoring system. Discrimination was quantitatively analyzed using area under the receiver operating characteristic (ROC) curve (AUC), and Calibration Curve. *p* < 0.05 were considered statistically significant.

## Results

### Procedural techniques and patient population in the training cohort

Between May 2015 and December 2021, 9841 patients received PCI treatment in the Department of Cardiology/Coronary Artery Center, Beijing AnZhen Hospital. 1508 total patients with CTO were treated by PCI at our hospital. Of these patients, 309 total patients underwent retrograde PCI for CTO recanalization (1199 patients were treated via antegrade approach only). They were divided into a training cohort (208 patients with 348 collateral channels) and a validation cohort (101 patients with 115 collateral channels).

In the training cohort, of the 208 patients, 95 (45.7%) patients previously failed antegrade approach attempt. Among them, 12 failed antegrade attempts occur in the current hospitalization, and 83 occur in other hospitals. In the remaining 113 (54.3%) patients, the CTO-PCI has never been attempted before and the retrograde approach was selected at the operator’s discretion. In the 208 patients, guidewires traversed the collateral channels successfully in 104 patients (50.0%) of the 208 retrograde PCI patients. Microcatheters failed to traverse collateral channel circulation with guidewire in 2 patients. For the 2 patients, both Corsair and Finecross microcatheters were used and the small balloon inflation were attempted, but microcatheters still did not traverse the collateral channels. The PCI-CTO procedure is successful without any complication in 78 patients (Fig. 2). In the current study, we use 348 collateral channels attempted in the PCI, not 208 CTO patients as analysis objects.

### Age < 65 and LVEF ≥ 50% are independent predictors of successful retrograde collateral channel traverse

The demographic and clinical parameters from the 208 patients (Table 1) and their collateral channels (Table S1) were collected and analyzed. Gender, high BMI, smoking history, hypertension, hyperlipemia, diabetes, medical history, and previous PCI history were not statistically significantly related upon successful collateral channel traverse, confirmed by univariable regression analyses (Table 2). However, the collateral channel traverse success rate increased significantly in patients < 65 years old, compared to those older than 65 years (*p* < 0.001). Additionally, the collateral channel traverse success rate in the patient with LVEF ≥ 50 is significant higher in those with LVEF < 50% (*p* = 0.016), confirmed by univariable regression analyses (*p* < 0.001).

**Table 1:** Baseline patient demographic characteristics

	**Patients with collateral channel traverse success** **(*n* = 104)**	**Patients with collateral channel traverse failure** **(*n* = 104)**	**Total**	***p* value**
**Gender, n (%)**				
Male	88(84.6)	90(86.5)	178(85.6)	0.693
Female	16(15.4)	14(13.5)	30(14.4)	
**Age, n (%)**				** < 0.001**
< 65yrs	79(76.0)	29(27.9)	108(51.9)	
≥ 65yrs	25(24.0)	75(72.1)	100(48.1)	
**BMI, n (%)**				0.253
< 24 kg/m^2^	21(21.9)	29(29.0)	50(25.5)	
≥ 24 kg/m^2^	75(78.1)	71(71.0)	146(74.5)	
**Current and past smokers, n (%)**	52(50.0)	54(51.9)	106(51.0)	0.781
**Comorbidities, n (%)**				
Hypertension	61(58.7)	71(68.3)	132(63.5)	0.150
Hyperlipemia	39(38.6)	30(28.8)	69(33.7)	0.139
Diabetes mellitus	44(42.3)	35(33.7)	79(38.0)	0.199
**Medicine use before PCI, n (%)**				
ACEI/ARB,	55(52.9)	51(49.0)	106(51.0)	0.579
ß-blocker	83(79.8)	75(72.1)	158(76.0)	0.194
CCB	25(24.0)	55(52.9)	80(38.5)	** < 0.001**
Nitrates	86(82.7)	87(83.7)	173(83.2)	0.853
**LVEF, n (%)**				**0.016**
< 50%	11(10.6)	24(23.1)	35(16.8)	
≥ 50%	93(89.4)	80(76.9)	173(83.2)	
**Previous PCI history**	55(52.9)	59(56.7)	114(54.8)	0.577

Categorical variables were presented as number (percentage). P values were calculated using analysis of variance. Chi-square including Fisher’s exact tests were used to compare differences in variables between collateral channel traverse success and failure groups

*Abbreviation*: *BMI* body mass index, *PCI* percutaneous coronary intervention, *ACEI* angiotensin-converting enzyme inhibitor, *ARB* angiotensin receptor blocker, *CCB* calcium channel blocker, *LVEF* left ventricular injection fraction

**Table 2:** The results of univariable analyses for successful collateral channel traverse

	**OR**	**95% CI**	***p* value**
**Demographic characteristics**			
Gender (male)	1.360	0.754–2.755	0.307
Age (< 65 yrs)	1.714	1.085–2.710	**0.021**
BMI (≥ 24 kg/m^2^)	1.156	0.701–1.907	0.569
Past & current smokers	0.885	0.580–1.350	0.571
Hypertension	1.268	0.821–1.961	0.285
Hyperlipidemia	1.370	0.862–2.178	0.183
Diabetes mellitus	1.149	0.739–1.786	0.537
Medicine before PCI			
ACEI/ARB	1.116	0.731–1.704	0.610
ß-blocker	1.552	0.931–2.587	0.092
CCB	1.461	0.930–2.295	0.100
Nitrates	1.029	0.564–1.876	0.927
LVEF (LVEF ≥ 50%)	3.319	1.705–6.459	** < 0.001**
Previous PCI history	0.779	0.510–1.190	0.248
**CTO lesion characteristics**			
Targeted vessel			
LCX (Ref. LAD)	1.400	0.245–7.997	0.705
RCA (Ref. LAD)	1.810	0.484–1.236	0.283
In-stent occlusion	1.273	0.594–2.731	0.535
Morphology of entry point			
Ambiguous (Ref. Clear)	0.700	0.414–1.182	0.182
Invisible (Ref. Clear)	0.931	0.561–1.545	0.782
Calcification of lesion	0.589	0.343–1.011	0.055
Occluded route bending	1.410	0.922–2.156	0.112
Proximal cap side-branch	1.225	0.592–2.536	0.584
**Collateral channel characteristics**			
Collateral channels types (epicardial)	8.970	0.970–85.522	0.057
CC score			
CC 1 (Ref. CC0)	1.519	0.502–1.519	**0.460**
CC 2 (Ref. CC0)	6.107	1.634–22.820	**0.007**
Rentrop score			
1 (Ref. 0)	0.095	0.013–0.690	**0.020**
2 (Ref. 0)	5.495	1.869–16.158	**0.002**
3 (Ref. 0)	0.237	0.042–1.343	0.104
Connection between collateral channel and receptor vessels (clear)	7.645	4.772–12.471	** < 0.001**
Angle between donor vessels and collateral channels (< 90°)	0.272	0.055–1.339	0.109
Angle between receptor vessels and collateral channels (< 90°)	0.595	0.249–1.421	0.242
Channel tortuosity(mild)	1.381	1.005–2.412	**0.041**

The ORs, 95%CI, and *P*-value of the variables in demographic and CTO lesion characteristics were derived from the univariable logistic regression. The ORs, 95%CI, and *P*-value of the variables in collateral channel characteristics were derived from the regression model estimated by Generalized Estimating Equations

*Abbreviation*: *OR* odds ratios, *CI* confidence intervals, *BMI* body mass index, *PCI* percutaneous coronary intervention, *ACEI* angiotensin-converting enzyme inhibitor, *ARB* angiotensin receptor blocker, *CCB* calcium channel blocker, *LVEF* left ventricular injection fraction, *CTO* chronic total occlusion, *LAD* left anterior descending artery, *LCX* left circumflex artery, *RCA* right coronary artery

### CTO lesion was not associated with successful retrograde collateral channels traverse

Table 3 summarized the angiographic anatomic characteristics of the CTO lesion in the 348 collateral channels (including targeted vessels type, In-stent Occlusion, Morphology of the entry point, calcification of lesion, occluded route bending, proximal cap side-branch and occluded period). In antegrade PCI approach technique, these characteristics are crucial. Heretofore, it remains unknown whether such characteristics bear significance upon retrograde collateral channel traverse. We identified that there was no significant relationship between CTO lesion characteristics and successful retrograde collateral traverse, confirmed by univariable regression analyses (Table 2).

**Table 3:** Baseline angiographic characteristics of CTO lesion

	**Collateral channeltraverse success** **(*n* = 190)**	**Collateral channelstraverse failure** **(*n* = 158)**	**Total**	***p* value**
**Targeted vessel, n (%)**				
LAD	60 (31.6)	42 (26.6)	102 (29.3)	0.469
RCA	126 (66.3)	114 (72.1)	240 (69.0)	
LCX	4 (2.1)	2 (1.3)	6 (1.7)	
**In-stent occlusion, n (%)**	18 (9.5)	12 (7.6)	30 (8.6)	0.534
**Morphology of entry point, n (%)**				0.395
Tapered	85 (46.2)	65 (41.1)	150 (43.9)	
Blunt	43 (23.4)	47 (29.7)	90 (26.3)	
Invisible	56 (30.4)	46 (29.2)	102 (29.8)	
**Calcification of lesion, n (%)**	29 (15.3)	37 (23.4)	66 (19.0)	0.053
**Occluded route bending, n (%)**				0.112
< 45˚	110 (57.9)	78 (49.4)	188 (54.0)	
≥ 45˚	80 (42.1)	80 (50.6)	160 (46.0)	
**Proximal cap side-branch, n (%)**	174 (91.6)	142 (89.9)	316 (90.8)	0.584
**Occluded period, n (%)**				
3 months – 12 months	64 (43.5)	60 (45.8)	124 (44.6)	0.705
≥ 12 months	83 (56.5)	71 (54.2)	154 (55.4)	

Categorical variables were presented as number (percentage). *P* values were calculated using analysis of variance. Chi-square including Fisher’s exact tests were used to compare differences in variables between collateral channel traverse success and failure groups

*Abbreviation*: *LAD* left anterior descending artery, *RCA* right coronary artery, *LCX* left circumflex artery

### Advanced CC grade, advanced Rentrop grade, and clear connections associated with successful retrograde collateral channels traverse

Within 348 collateral channels effectively attempted, 190 were successful and 158 failed. As it was demonstrated in Table 4, compared to septal collateral channels, the successful traverse ratio in epicardial vessels was significantly greater (*p* = 0.002). There was no relationship between the angles between donor (or recipient) vessels/collateral channels and successful retrograde collateral channel traverse. The successful rate of collateral channel traverse is higher in the higher grade of CC subgroup (CC0 35.4%, CC1 70.8%, and CC2 75.8%, respectively, *p* < 0.001). In the univariable analysis, the successful rates of collateral channel traverse showed significant difference between CC0 and CC2 (*p* = 0.007). Whereas it showed no significant difference between CC0 and CC1 (*p* = 0.460). Similarly, the successful rate of collateral channel traverse is higher in the higher grade of Rentrop subgroup (Rentrop 0 and 1 28.4%, Rentrop 2 and 3 61.1%, respectively, *p* < 0.001). There was no significant difference between Rentrop grades 2 and 3. Connections between collateral channels and recipient vessels were vital in successful retrograde collateral circulation traverse. When Connections were clear, the collateral traverse ratio was significantly increased (77.2%). When the connections were ambiguous or invisible, the collateral traverse ratio was 30.7%. Collateral channel continuity was a property more crucial than vessel diameter in the successful retrograde collateral circulation traverse. In addition, collateral tortuosity exerted the significant effects on collateral traverse. Compared with mild tortuosity (59.7%), severe tortuosity (48.4%) resulted in the significant decreasing in successful collateral traverse (*p* = 0.039).

**Table 4:** Baseline angiographic characteristics of collateral channels

	**Collateral channel traverse success** **(*n* = 190)**	**Collateral channel traverse failure** **(*n* = 158)**	**Total**	***p* value**
**Collateral channels types, n (%)**				**0.002**
Septal	150 (78.9)	144 (91.1)	294 (84.5)	
Epicardial	40 (21.1)	14 (8.9)	54 (15.5)	
**CC score, n (%)**				** < 0.001**
0	58 (30.5)	106 (68.0)	164 (47.4)	
1	85 (44.7)	35 (22.4)	120 (34.7)	
2	47 (24.8)	15 (9.6)	62 (17.9)	
**Rentrop score, n (%)**				
0	7 (3.7)	15 (9.6)	22 (6.3)	** < 0.001**
1	12 (6.3)	33 (21.0)	45 (13.0)	
2	150 (78.9)	72 (45.9)	222 (64.0)	
3	21 (11.1)	37 (23.6)	58 (16.7)	
**Connection between collateral channel and receptor vessels, n (%)**				** < 0.001**
Clear	139 (73.2)	41 (26.3)	180 (52.0)	
Ambiguous/invisible	51 (26.8)	115 (73.7)	166 (48.0)	
**Angle between donor vessels and collateral channels, n (%)**				0.104
< 90˚	173 (91.1)	149 (95.5)	322 (93.1)	
≥ 90˚	17 (8.9)	7 (4.5)	24 (6.9)	
**Angle between receptor vessels and collateral channels, n (%)**				0.719
< 90˚	153 (87.4)	111 (88.8)	264 (88.0)	
≥ 90˚	22 (12.6)	14 (11.2)	36 (12.0)	
**Channel tortuosity, n (%)**				**0.039**
Mild	114 (60.6)	77 (49.4)	191 (55.5)	
Severe	74 (39.4)	79 (50.6)	153 (44.5)	

Categorical variables were presented as number (percentage). *P* values were calculated using analysis of variance. Chi-square were used to compare differences in variables between collateral channel traverse success and failure groups

### Predictors entering the scoring system

**Fig. 3:**
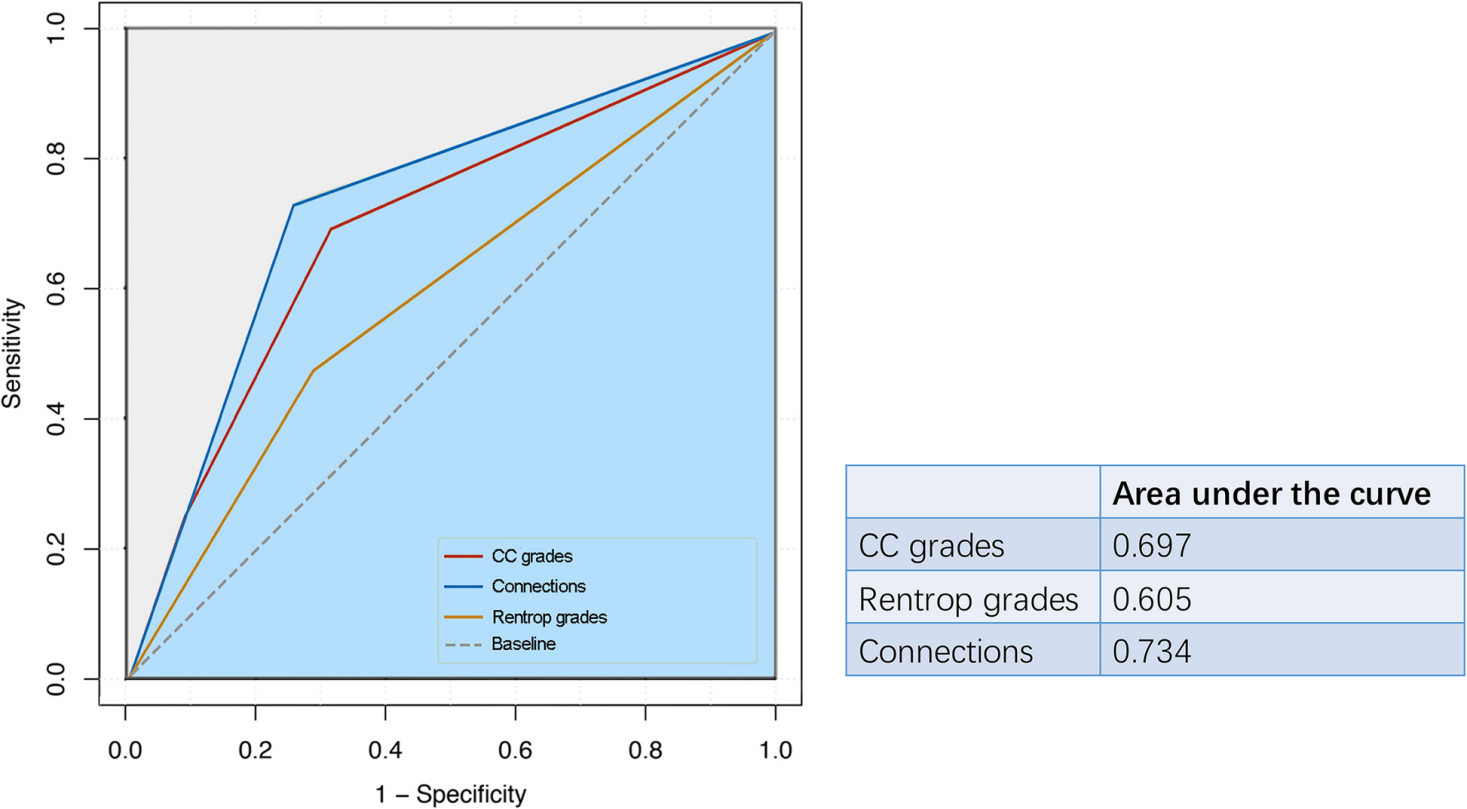
Area under the curve among CC grades, Rentrop grades, and the connections between collateral channels and recipient vessels

Based on the results from univariable regression and Generalized Estimating Equations analysis in Table 2, age, LVEF, CC grades, Rentrop grades, Connection, and collateral tortuosity were selected as the potential predictors. Among them, there was a similarity in the clinical significance of CC grades, Rentrop grades, and the Connection in terms of the collateral channel continuity. To evaluate the effectiveness of these three factors, we utilized the ROC Curve. Figure 3 illustrates that the area under the ROC Curve was largest for Connection (0.734) compared to CC grades (0.697) and Rentrop grades (0.605). Therefore, we incorporated Connection into the predictive scoring system, replacing CC grades and Rentrop grades.

Consequently, age, LVEF, Connection, and collateral tortuosity were subjected to multivariable regression analysis. The results from multivariable analyses indicated that all above factors showed significance upon retrograde collateral channel traverse (Table 5). However, LVEF variable was excluded in the nomogram because the cases with LVEF ≤ 50 in the collateral channel traverse success group was less than 5 (*n* < 5) after deleting missing data, leading to a substantial deviation and potentially affecting the model's stability. Thus, the final variables included in the nomogram are age, Connection, and collateral tortuosity.

**Table 5:** The results of multivariable analyses for successful collateral traverse

	**Crude OR**	**95%CI**	**Adjusted OR**	**95% CI**	***P*** **value**
**Age (< 65 yrs)**	1.714	1.085–2.710	1.960	1.138–3.377	0.015
**LVEF (LVEF > 50%)**	3.319	1.705–6.459	5.966	1.797–19.801	0.004
**Connections (clear)**	7.645	4.772–12.471	8.596	5.174–14.282	< 0.001
**Channel tortuosity** (**mild)**	1.581	1.005–2.412	2.091	1.259–3.475	0.004

The ORs, 95%CI, and *P*-value of the variables were derived from the multivariable logistic regression

*Abbreviation*: *OR* odds ratios, *CI* confidence intervals, *LVEF* left ventricular ejection fraction

### Establishment of the scoring system

**Fig. 4:**
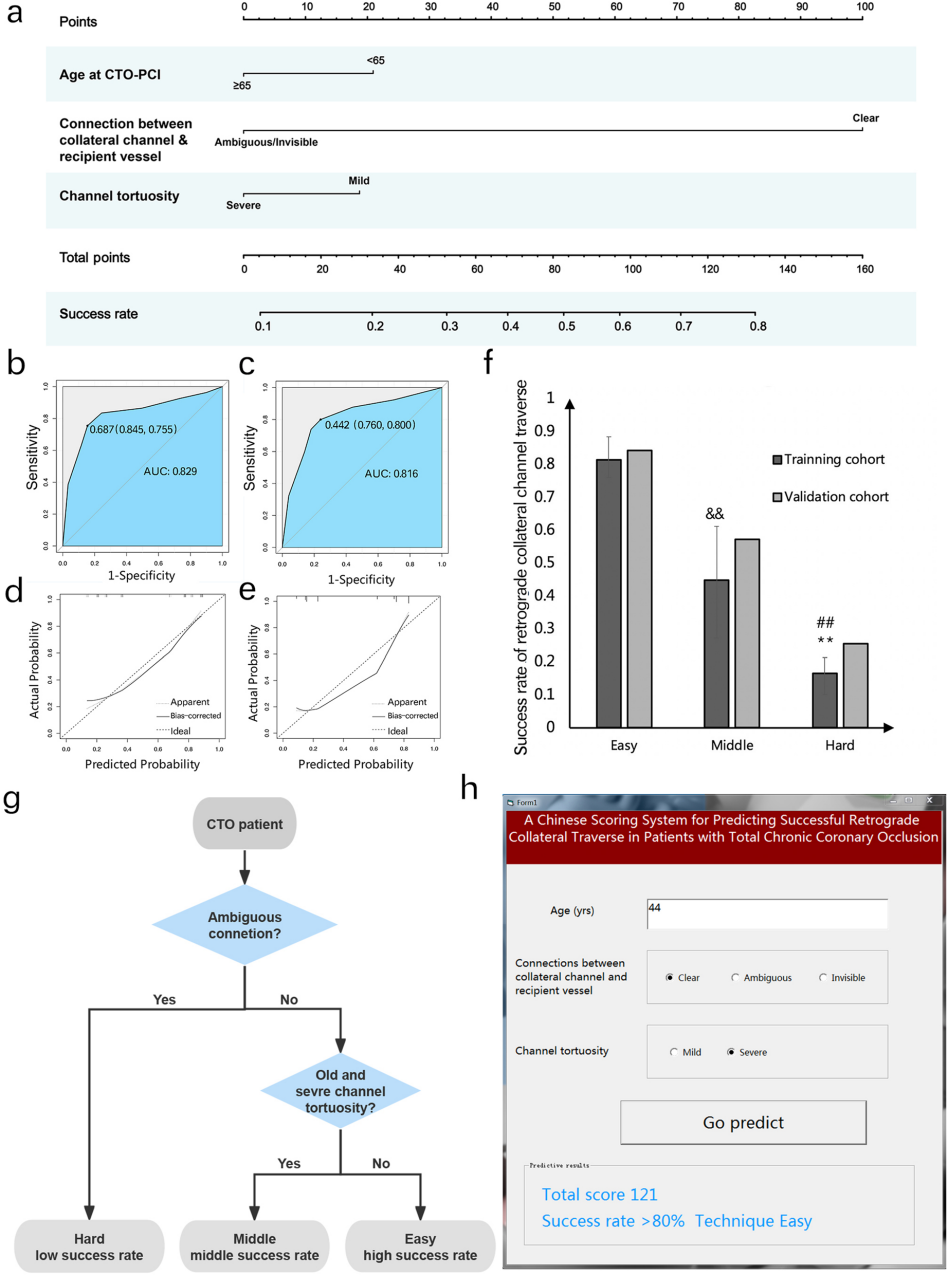
Nomogram and its efficiency: **a** A nomogram predictive scoring system for successful retrograde collateral channel traverse: age (21 point), connection between collateral channels and recipient vessels and collateral tortuosity (100 point), and channel tortuosity (19 points); **b** Scoring system verification: ROC of training cohort; **c** ROC of validation cohort; **d** The calibration curve in training cohort; and **e** The calibration curve in validation cohort; **f** The success rate in Easy, Middle, and Hard group, showing the significant discrimination; **g** The workflow for quick evaluation in clinical practice; **h** The interface of the ACT scoring system software

Accordingly, a predictive scoring system for successful retrograde collateral channel traverse was developed on the basis of the aforementioned 3 risk predictors: age (21 point), Connections (100 point), and channel tortuosity (19 points). As the nomogram shown in Fig. 4a, for example, in a 46-year-old CTO patient (21 point), coronary artery angiography showed a mild channel tortuosity (19 points) but an ambiguous/invisible connection between collateral channel and recipient vessel (0 points). Thus, the total score is 40 points, which means there only 24% rate in successful retrograde collateral traverse by guide wire.

### Scoring system verification

Internal verification in the training cohort showed that the AUC was 0.826. The sensitivity and specificity of the ROC curve were 0.755 and 0.845, respectively. The optimal cutoff was 0.687 according to the ROC curve. The baseline characteristic of training cohort and validation cohort showed no significant differences except the variable of target vessel. The percentage of target vessel LCX was significantly higher than that in the validation cohort (*p* = 0.004). The predictors selected in the nomogram model (age, Connections, and channel tortuosity) in this two cohort showed no significant difference (*p* = 0.215, 0.894, and 0.343, respectively) (Table S2). In the validation cohort for external verification, the AUC was 0.816 with 0.800 and 0.760 in sensitivity and specificity, respectively, which showed good concordance (Fig. 4b, c). The calibration curve using bootstrapping (resampling = 2000 times) showed that there was also good concordance between the predicted and observed values of success rate of retrograde collateral channel traverse by guidewire in both training and validation cohorts (Fig. 4d, e). 

### Risk categories and software for the quick clinical evaluation

In order to make convenient assessment of success rate of collateral traverse in the PCI for the clinical physician, we divided the retrograde technique level into three groups based on the risk score generated in the ROC analysis: Easy (high success rate category, risk score > 0.66), Middle (middle success rate category, risk score 0.33–0.66), and Hard(low success rate category, risk score < 0.33). In the training cohort, the success rate is 81.5%, 44.8%, and 16.5% in Easy, Middle, and Hard group respectively, showing the significant discrimination (*p* < 0.001). And it showed a consistent prediction power in the validation cohort (Fig. 4f). After analysis the predictors component in each category, we summarized the workflow for quick evaluation in clinical practice (Fig. 4g). We have also developed a scoring system software (.exe form, as Supplemental materials), which is compatible with the Windows operating system. This software incorporates the nomogram weight coefficient score for each predictor in the model. When the values of the predictors are inputted into the software interface, it can generate the total score of this scoring system, the predictive success rate, and an evaluation of the technique difficulty (Fig. 4h). The software might enhance the convenience of using the scoring system in clinical practice.

## Discussion

The retrograde approach to CTO PCI is an important complement to the classic antegrade approach, especially in cases where the proximal cap is poorly visualized, or in challenging lesions where the antegrade approach has failed [10, 17]. Absence of “interventional” collaterals (vessels suitable for retrograde approach) deprives the operator of alternative traverse strategy and eventually contributes to technical failure probability [18, 19]. Our study sought to identify factors related to successful retrograde collateral circulation traverse of CTO patient lesions in the setting of suitable collateral presence.

The present study demonstrated that age and collateral morphology are significant factors influencing successful retrograde collateral traverse likelihood. Regarding morphologic characteristics, vessel continuity and tortuosity (not diameter) are related to successful retrograde collateral traverse. Most important is the presence of a continuous connections between collaterals and recipient vessels. For a collateral vessel to be suitable for the retrograde procedure, the connection must be clear [20]. In the present study, operators often sought thick collaterals (at their entry point) as potentially viable vessels to traverse. However, some vessels that were thick at entry-point were not continuous between collateral and recipient vessels, resulting in traverse failure. In contrast, traverse attempts were successful in minute collaterals, which were continuous and clear. In our study, age is an independent predictive factor for predicting collateral channel traverse. The success rate of CTO-PCI procedures is closely associated with age, as several previous studies have indicated that younger patients have higher rates of procedural technical success compared to older patients [21–25]. According to a comprehensive review of the literature, Technique success was reported by summarizing previous studies and it showed that CTO-PCI was successful in 78.1% younger patients and 82.8% in older patients [26]. The underlying mechanism lies in that elderly patients are more likely to have complex coronary artery lesions, poor collateralization. The previous study identified that the prevalence of well-developed collateral circulation in the younger group (≤ 64 years) was significantly higher than that in the older group (≥ 65 years) [27]. This combined with the comorbidity burden accounts for subsequent limited attempts of physicians towards performing retrograde technique in the high-risk group. Heart failure(LVEF < 50) was also identified as an important independent predictive factor for collateral channel traverse failure, and its underlying mechanism lies in the fact that heart failure, especially heart failure with reduced ejection fraction, leads to a decrease in coronary artery perfusion pressure, resulting in unclear visualization of the entry points and collateral channels. Furthermore, patients with heart failure exhibit decreased tolerance to myocardial ischemia and diminished compensatory ability. When implementing a retrograde approach, they are more prone to hemodynamic instability, leading operators to adopt a more conservative approach.

**Fig. 5:**
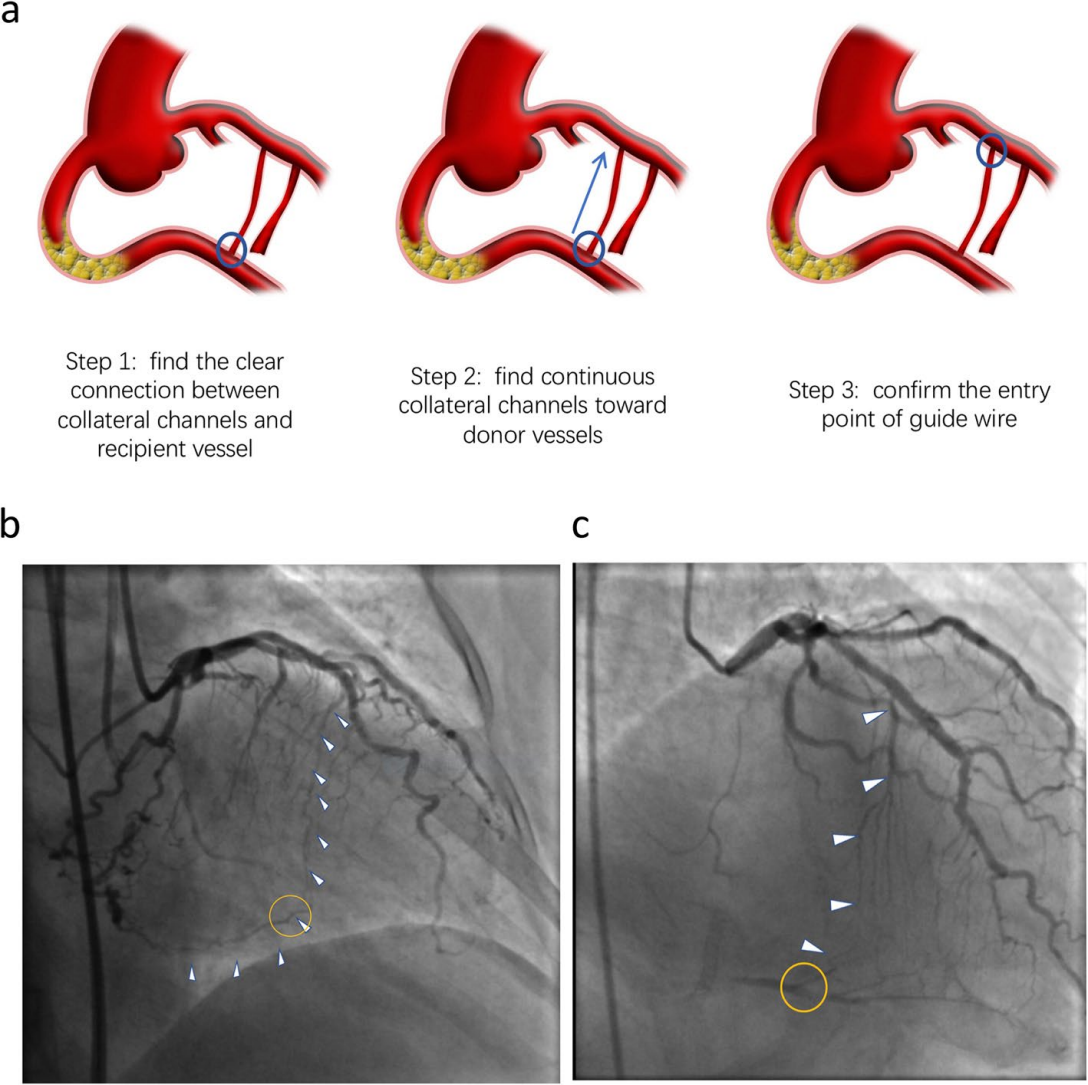
Our recommendation for retrograde collateral circulation traverse approach: **a **The steps for retrograde collateral circulation traverse; **b** and **c **The original angiographic pictures of collateral selection. Orange circle indicated connection between collateral and recipient vessels; white arrows pointed to the selected channels

Figure 5a reveals our center’s retrograde collateral circulation traverse approach. Firstly, a clear connection must be identified between collateral and recipient vessels. Secondly, continuous collaterals must be identified via retrograde approach. Thirdly, a patent entry point between donor and collateral vessels should be evaluated. Finally, the guidewire should traverse the collaterals via preselected collateral channels. As collateral vessel diameter decreases gradually from donor to recipient vessels, it is imperative that the connections between collateral and recipient vessels be clear. The original angiographic pictures were shown in Fig. 5b, c. Connections in orange circle is clear. Although the selected channels were tiny, guidewires traversed these channels successfully.

Two general collateral circulation traverse approaches exist. The original Japanese method involves selecting a collateral vessel with high probability of connection, reliance upon sequential distal microcatheter tip injections for visualization and isolation of the connecting collateral vessel, and attempted traverse with a dedicated wire [5, 12, 28–30]. The Western method, also known as the “septal surfing technique” (SST, first described by Sianos, forgoes distal tip injections and employs wire probing via trial and error in seeking a path of lowest resistance in the septal collateral channels [28].

Few studies have evaluated predictors of retrograde CTO PCI success [12, 31–33]. A small single center Japanese study of 157 patients included 106 septal collateral circulation vessels and reported that the absence of any clear connection (CC grade 0) was a predictor of failure, supported by our present study [34]. We regard visualization as being vital to successful wire traverse of the collateral circulation. We further demonstrate that connections between collateral channels and recipient vessels were vital in successful retrograde collateral channel traverse. When such connections were clear, the collateral traverse ratio was significantly increased. The fact that invisible septals were crossed with as much success as larger collateral vessels, merits some discussion. In the Western methods, CC0 collateral channels sometimes might be selected as target vessels [35]. In the present study, successful traverse is much significantly decreased in invisible collateral vessels (CC grade 0, Rentrop grades 0 or 1). We compared the results between present study and a study from Canada (Dautov R et al.) [36]. In their procedure, septal surfing was used, and interrupted collaterals were selected. However, as should be mention, the perforation ratio is significant higher during their procedures, as shown in Table 6. Such a complication can potentially lead to emergency surgery or even death [37]. We also explored the complication rates between septal channel and epicardial channel types, the complications rates including collateral perforation, side branch occlusion, donor vessel dissection, and recipient vessel dissection, all showed no significant difference in septal and epicardial channels (Table S3). Therefore, we inferred that selecting clear and continuous channels instead of specific channel types might be safer during collateral traverse.

**Table 6:** The comparison of collateral perforation ratio between Dautov R’ and our study

	**Total collateral channels, n**	**Collateral perforation, n (%)**	***p***
**Dautov R’ study**^a^	240	45 (18.8)	< 0.001
**Our study**	348	10 (2.9)	

^a^Dautov R, Urena M, Nguyen CM, Gibrat C, Rinfret S (2017) Safety and effectiveness of the surfing technique to cross septal collateral channels during retrograde chronic total occlusion percutaneous coronary intervention. EuroIntervention 12(15):e1859-e67. https://doi.org/10.4244/EIJ-D-16-00650

In summary, based upon 309 retrograde CTO-PCI cases involving 463 collateral channels traverse attempted in PCI, the presence of clear connections between collateral and recipient vessels predicts whether guidewire may successfully traverse the collateral circulation. Additionally, age and collateral tortuosity bear significance on successful collateral traverse. Our results may be of potential value to worldwide interventional cardiovascular providers in the treatment of CTO patients.

As our study was a retrospective observational study, limitations inherent to such a design study are applicable. The cases were selected from single center. Therefore, the selection deviation might occur. And the use of medication might be affected by the recall deviation. Additionally, this was a single center study involving five experienced operators (more than 10 years interventional experiences). The generalizability of our data may therefore be limited to similar institutions employing similarly experienced operators.

## Conclusions

The novel Chinese ACT score is a reliable tool for predicting successful retrograde collateral traverse.

## Supplementary Information

**Additional file 1:** **Table. S1.** Baseline collateral channel demographic characteristics.

**Additional file 2:** **Table S2. **The demographic baseline of patients between training cohort and validation cohort.

**Additional file 3:** **Table S3. **The complication of retrograde CTO-PCI in septal and epicardial collateral channels.

**Additional file 4.** ACT scoring system software.

## Data Availability

The datasets used and/or analyzed during the current study are available from the corresponding author on reasonable request.

## Abbreviations

ACT: The name of the scoring system for predicting successful collateral channels traverse via retrograde approach, which is formed by initials of Age, Connections between collateral channels and recipient vessels, and Channel Tortuosity
AUC: Area under curve
BMI: Body mass index
Connections: connections between collateral channels and recipient vessels
CTO: Chronic total occlusion
LVEF: Left ventricular ejection fraction
PCI: Percutaneous coronary intervention
ROC: Receiver operating characteristic
