# Supplementary material for: A Chinese scoring system for predicting successful retrograde collateral traverse in patients with chronic total coronary occlusion
Source: BMC Cardiovasc Disord. 2023 Jul 29;23:380. doi: 10.1186/s12872-023-03405-6 (PMC10386207; doi:10.1186/s12872-023-03405-6)
Supplement: Supplementary file 1 — Additional file 1: Table. S1. Baseline collateral channel demographic characteristics. [file 12872_2023_3405_MOESM1_ESM.docx]

**Table. S1 Baseline collateral channel demographic characteristics**

|  | Collateral channel traverse success  (n=190) | Collateral channel traverse failure  (n=158) | Total | *p* value |
| --- | --- | --- | --- | --- |
| Gender, n (%) |  |  |  |  |
| Male | 165 (86.8) | 131 (82.9) | 296 (85.1) | 0.306 |
| Female | 25 (13.2) | 27(17.1) | 52 (14.9) |  |
| Age, n (%) |  |  |  | **<0.001** |
| <65yrs | 141 (74.2) | 49 (31.0) | 190 (54.6) |  |
| ≥65yrs | 49 (25.8) | 109 (69.0) | 158 (45.4) |  |
| BMI, n (%) |  |  |  | 0.569 |
| <24 kg/m^2^ | 46 (25.3) | 40 (26.3) | 82 (24.8) |  |
| ≥24 kg/m^2^ | 136 (74.4) | 112 (73.7) | 248 (75.2) |  |
| Current and past smokers, n (%) | 102 (53.7) | 80 (50.6) | 182(52.3) | 0.570 |
| Comorbidities, n (%) |  |  |  |  |
| Hypertension | 112 (58.9) | 102 (64.6) | 214 (61.5) | 0.284 |
| Hyperlipemia | 63 (33.9) | 43 (27.2) | 106 (30.8) | 0.183 |
| Diabetes Mellitus | 71 (37.4) | 54 (34.2) | 125 (35.9) | 0.537 |
| Medicine use before PCI, n (%) |  |  |  |  |
| ACEI/ARB, | 93 (48.9) | 73 (46.2) | 166 (47.7) | 0.610 |
| ß-blocker | 155 (81.6) | 117 (74.1) | 272 (78.2) | 0.091 |
| CCB | 54 (28.4) | 74 (46.8) | 128 (36.8) | 0.135 |
| Nitrates | 163 (85.8) | 135 (85.4) | 298 (85.6) | 0.927 |
| LVEF, n (%) |  |  |  | **<0.001** |
| <50% | 14 (7.4) | 33 (20.9) | 47 (13.5) |  |
| ≥50% | 176 (92.6) | 125 (79.1) | 301(86.5) |  |
| Previous PCI history | 94 (49.5) | 88 (55.7) | 182 (52.3) | 0.247 |

Categorical variables were presented as number (percentage). P values were calculated using analysis of variance. Chi-square including Fisher’s exact tests were used to compare differences in variables between collateral channel traverse success and failure groups. Abbreviation: BMI, body mass index; PCI, percutaneous coronary intervention; ACEI, angiotensin-converting enzyme inhibitor; ARB, angiotensin receptor blocker; CCB, calcium channel blocker; LVEF, left ventricular injection fraction.
