# Supplementary material for: A Chinese scoring system for predicting successful retrograde collateral traverse in patients with chronic total coronary occlusion
Source: BMC Cardiovasc Disord. 2023 Jul 29;23:380. doi: 10.1186/s12872-023-03405-6 (PMC10386207; doi:10.1186/s12872-023-03405-6)
Supplement: Supplementary file 2 — Additional file 2: Table S2. The demographic baseline of patients between training cohort and validation cohort. [file 12872_2023_3405_MOESM2_ESM.docx]

**Table S2. The demographic baseline of patients between training cohort and validation cohort**

|  | Training cohort  (n=208) | Validation cohort  (n=101) | Total  (n=309) | *p* value |
| --- | --- | --- | --- | --- |
| Gender, n (%) |  |  |  |  |
| Male | 178 (85.6) | 87 (86.1) | 265 (85.8) | 0.895 |
| Female | 30 (14.4) | 14 (13.9) | 44 (14.2) |  |
| Age, n (%) |  |  |  |  |
| <65yrs | 108 (51.9) | 60 (59.4) | 168 (54.4) | 0.215 |
| ≥65yrs | 100 (48.1) | 41(40.6) | 141 (45.6) |  |
| BMI, n (%) |  |  |  |  |
| <24 kg/m^2^ | 50 (25.5) | 27 (26.7) | 77(25.9) | 0.820 |
| ≥24 kg/m^2^ | 146 (74.5) | 74 (73.3) | 220 (74.1) |  |
| Current and past smokers, n (%) | 106 (51.0) | 46 (45.5) | 152 (49.2) | 0.372 |
| Comorbidities, n (%) |  |  |  |  |
| Hypertension | 132 (63.5) | 69 (68.3) | 201 (65.0) | 0.401 |
| Hyperlipemia | 69 (33.7) | 29 (28.7) | 98 (32.0) | 0.383 |
| Diabetes mellitus | 79 (38.0) | 43 (42.6) | 122 (39.5) | 0.483 |
| Medicine use before PCI, n (%) |  |  |  |  |
| ACEI/ARB | 106(51.0) | 63(62.4) | 169 (54.7) | 0.059 |
| ß-blocker | 158(76.0) | 70(69.3) | 228 (73.8) | 0.212 |
| CCB | 80(38.5) | 48(47.5) | 128 (41.4) | 0.129 |
| Nitrates | 173(83.2) | 84(83.2) | 257 (83.2) | 0.999 |
| LVEF, n (%) |  |  |  |  |
| <50% | 35(16.8) | 21(20.8) | 56 (18.1) | 0.396 |
| ≥50% | 173(83.2) | 80(79.2) | 253 (81.9) |  |
| Previous PCI history | 114(54.8) | 65(64.4) | 179 (57.9) | 0.111 |

|  | Collateral channels in the training cohort  (n=348) | Collateral channels in the validation cohort  (n=115) | Total  (n=463) | *p* value |
| --- | --- | --- | --- | --- |
| Targeted Vessel, n (%) |  |  |  |  |
| LAD | 102 (29.3) | 35 (30.4) | 137 (29.6) | **0.004** |
| RCA | 240 (69.0) | 69 (60.0) | 309 (66.7) |  |
| LCX | 6 (1.7) | 11 (9.6) | 26 (5.6) |  |
| In-stent occlusion, n (%) | 30 (8.6) | 14(13.9) | 44(9.5) | 0.260 |
| Morphology of entry point, n (%) |  |  |  |  |
| Tapered | 150 (43.9) | 45 (39.1) | 195 (42.7) | 0.665 |
| Blunt | 90 (26.3) | 32 (27.8) | 122 (26.7) |  |
| Invisible | 102 (29.8) | 38 (33.1) | 140 (30.6) |  |
| Calcification of lesion, n (%) | 66 (19.0) | 19 (16.5) | 85 (18.4) | 0.557 |
| Occluded route bending, n (%) |  |  |  |  |
| <45˚ | 188 (54.0) | 63 (54.8) | 251 (54.2) | 0.887 |
| ≥45˚ | 160 (46.0) | 52 (45.2) | 212 (45.8) |  |
| Proximal cap side-branch, n (%) | 316 (90.8) | 102 (88.7) | 418 (90.3) | 0.508 |
| Collateral channels types, n (%) |  |  |  | 0.184 |
| Septal | 294 (84.5) | 91 (79.1) | 385 (83.2) |  |
| Epicardial | 54 (15.5) | 24 (20.9) | 78 (16.8) |  |
| CC score, n (%) |  |  |  | 0.706 |
| 0 | 164 (47.4) | 58 (50.4) | 222 (48.2) |  |
| 1 | 120 (34.7) | 35 (30.4) | 155 (33.6) |  |
| 2 | 62 (17.9) | 22 (19.1) | 84 (18.2) |  |
| Rentrop score, n (%) |  |  |  | 0.937 |
| 0 | 22 (6.3) | 8 (7.0) | 30 (6.5) |  |
| 1 | 45 (13.0) | 17 (14.8) | 62 (13.4) |  |
| 2 | 222 (64.0) | 70 (60.9) | 292 (63.2) |  |
| 3 | 58 (16.7) | 20 (17.4) | 78 (16.9) |  |
| Connection between collateral channel and receptor vessels, n (%) |  |  |  | 0.894 |
| Clear | 180 (52.0) | 59 (51.3) | 239 (51.8) |  |
| Ambiguous/invisible | 166 (48.0) | 56 (48.7) | 222 (48.2) |  |
| Angle between donor vessels and collateral channels, n (%) |  |  |  | 0.226 |
| <90˚ | 322 (93.1) | 103 (89.6) | 425 (92.2) |  |
| ≥90˚ | 24 (6.9) | 12 (10.4) | 36 (7.8) |  |
| Angle between receptor vessels and collateral channels, n (%) |  |  |  | 0.844 |
| <90˚ | 264 (88.0) | 102 (88.7) | 366 (88.2) |  |
| ≥90˚ | 36 (12.0) | 13 (11.3) | 49 (11.8) |  |
| Channel tortuosity, n (%) |  |  |  | 0.343 |
| Mild | 191 (55.5) | 58 (50.4) | 249 (54.2) |  |
| Severe | 153 (44.5) | 57 (49.6) | 210 (45.8) |  |

Categorical variables were presented as number (percentage). P values were calculated using analysis of variance. Chi-square were used to compare differences in variables between training and validation groups. Abbreviation: BMI, body mass index; PCI, percutaneous coronary intervention; ACEI, angiotensin-converting enzyme inhibitor; ARB, angiotensin receptor blocker; CCB, calcium channel blocker; LVEF, left ventricular injection fraction; CTO, chronic total occlusion; LAD, left anterior descending artery; LCX, left circumflex artery; RCA, right coronary artery.
