# Supplementary material for: A Chinese scoring system for predicting successful retrograde collateral traverse in patients with chronic total coronary occlusion
Source: BMC Cardiovasc Disord. 2023 Jul 29;23:380. doi: 10.1186/s12872-023-03405-6 (PMC10386207; doi:10.1186/s12872-023-03405-6)
Supplement: Supplementary file 3 — Additional file 3: Table S3. The complication of retrograde CTO-PCI in septal and epicardial collateral channels. [file 12872_2023_3405_MOESM3_ESM.docx]

**Table S3. The complication of retrograde CTO-PCI in septal and epicardial collateral channels**

| Complication | Septal | | |  | Epicardial | |  | P |
| --- | --- | --- | --- | --- | --- | --- | --- | --- |
|  | Training cohort(n=294) | Validation cohort(n=91) | Total (n=385) | | Training cohort(n=54) | Validation cohort(n=24) | Total (n=78) |  |
| Collateral perforation, n (%) | 8(2.7) | 3(3.3) | 11(2.9) | | 2(3.7) | 0(0) | 2(2.6) | 0.999 |
| Side branch occlusion, n (%) | 2(0.7) | 0(0) | 2(0.3) | | 1(1.9) | 1(4.2) | 2(2.6) | 0.133 |
| Donor vessels dissection, n (%) | 1(0.3) | 1(1.1) | 2(0.5) | | 1(1.9) | 0(0) | 1(1.3) | 0.426 |
| Recipient vessels dissection, n (%) | 7(2.4) | 3(3.3) | 10(2.6) | | 2(3.7) | 1(4.2) | 3(3.8) | 0.476 |

Categorical variables were presented as number (percentage). P values were calculated using analysis of variance. Fisher’s exact tests were used to compare differences in complications between septal and epicardial collateral channel.
